# SEOM-GETNE clinical guidelines for the diagnosis and treatment of gastroenteropancreatic and bronchial neuroendocrine neoplasms (NENs) (2022)

**DOI:** 10.1007/s12094-023-03205-6

**Published:** 2023-05-19

**Authors:** Jaume Capdevila Castillón, Teresa Alonso Gordoa, Alberto Carmona Bayonas, Ana Custodio Carretero, Rocío García-Carbonero, Enrique Grande Pulido, Paula Jiménez Fonseca, Angela Lamarca Lete, Angel Segura Huerta, Javier Gallego Plazas

**Affiliations:** 1 Servicio de Oncología Médica, Hospital Universitario Vall D’Hebron, Ps Vall d’Hebron, 119-129, 08035 Barcelona, Spain; 2 Servicio de Oncología Médica, Hospital Universitario Ramón y Cajal, Madrid, Spain; 3 Servicio de Oncología Médica, Hospital Universitario Morales Meseguer, Murcia, Spain; 4 Servicio de Oncología Médica, Hospital Universitario La Paz, Madrid, Spain; 5 Servicio de Oncología Médica, Hospital Universitario 12 de Octubre, Madrid, Spain; 6 Servicio de Oncología Médica, MD Anderson Cancer Center, Madrid, Spain; 7 Servicio de Oncología Médica, Hospital Universitario Central de Asturias, Oviedo, Spain; 8 Servicio de Oncología Médica, Hospital Universitario Fundación Jiménez Díaz, Madrid, Spain; 9 Servicio de Oncología Médica, Hospital Universitari I Politècnic la Fe, Valencia, Spain; 10 Servicio de Oncología Médica, Hospital General Universitario de Elche, Alicante, Spain

**Keywords:** Guidelines, Neuroendocrine, Diagnosis, Therapy

## Abstract

Neuroendocrine neoplasms (NENs) are a heterogeneous family of tumors of challenging diagnosis and clinical management. Their incidence and prevalence continue to rise mainly due to an improvement on diagnostic techniques and awareness. Earlier detection, along with steadfast improvements in therapy, has led to better prognosis over time for advanced gastrointestinal and pancreatic neuroendocrine tumors. The aim of this guideline is to update evidence-based recommendations for the diagnosis and treatment of gastroenteropancreatic and lung NENs. Diagnostic procedures, histological classification, and therapeutic options, including surgery, liver-directed therapy, peptide receptor radionuclide therapy, and systemic hormonal, cytotoxic or targeted therapy, are reviewed and discussed, and treatment algorithms to guide therapeutic decisions are provided.

## Incidence and epidemiology

Incidence of neuroendocrine neoplasms (NENs) is continuously increasing due to an improvement on diagnostic techniques and awareness. The incidence rate in gastroenteropancreatic (GEP) NEN is 3.56 new cases per 100,000, followed by lung NEN with 1.49/100.000 and unknown primary NEN with 0.84/100.000 [1]. The greatest increase in incidence has been reported in lung NEN, grade (G) 1, and in localized tumors [1]. However, between 10 and 20% of NEN represent high-grade neoplasms, mainly neuroendocrine carcinomas (NEC) whose most frequent site of origin is the lung [2]. Unfortunately, more than half of those patients are still being diagnosed with advanced disease [2].

An increase in prevalence rate has also been reported from 0.006% in 1993 to 0.048% in 2012 [1]. According to the primary tumor origin, the prevalence rate has a different pattern across world regions: in Europe, the most prevalent ones originate from the small intestine (SI) and pancreas [2, 3].

The median age at diagnosis is 60 years [2]. Approximately 20% of neuroendocrine tumors (NETs) are considered functioning due to different tumor-released peptides that define a particular clinical behavior and prognosis [1]. No clear risk factors have been defined related to NEN development, but around 5% of them appear in the context of hereditary predisposition syndromes, such as type 1 multiple endocrine neoplasia (MEN 1) [2].

An improvement in overall survival (OS) over time has also been described in NET due to an earlier stage at diagnosis and increase in treatment options. The 5-year OS rate for localized disease is reported between 83 and 97% (25–60% in NEC), for regional disease between 67 and 84% (9.2–28.5% in NEC), and for metastatic disease between 28 and 50% (10% in NEC) [4, 5]. Overall, rectal and appendiceal NET harbor the best prognosis [2]. However, in patients with metastatic disease, the best prognosis is reported in SI-NET followed by pancreatic NET (panNET) and gastric/colorectal with a 5-year OS rate of 69%, 50%, and 30%, respectively [1]. In lung NETs, atypical carcinoids (AC) harbor a worse prognosis compared with typical carcinoids (TC) with a 5-year OS rate of 69% and 93%, respectively [6]. Finally, the presence of carcinoid syndrome is associated with worse prognosis [7].

## Methodology

This guideline is based on a systematic review of relevant published studies and with the consensus of ten treatment expert oncologists from GETNE (Spanish Group of Neuroendocrine and Endocrine Tumors) and SEOM (Spanish Society of Medical Oncology), and an external review panel of two experts designated by SEOM. The Infectious Diseases Society of America-US Public Health Service Grading System for Ranking Recommendations in Clinical Guidelines has been used to assign levels of evidence and grades of recommendation.

## Diagnosis, staging, and risk assessment

### Histological diagnosis

The histopathological diagnosis of NENs relies on characteristic morphological features and on the demonstration of the neuroendocrine phenotype of tumor cells through the immunohistochemical detection of a panel of markers including at least synaptophysin and chromogranin A. Insulinoma-associated protein 1 (INSM1) has recently been proposed as a promising nuclear marker, since it is highly sensitive and specific for NENs, irrespective of origin and differentiation status (III, A). Immunohistochemistry can also be helpful for determining the primary site of metastatic NENs of occult origin. Although not entirely specific, intestinal origin is favored by CDX2, pancreatic origin by Islet-1 and/or PAX6, and lung origin by TTF-1 [8, 9] (III, B).

According to the most recent 2019 World Health Organization (WHO) Classification [10], GEP-NENs should be classified based on morphology and grade (assessed using either Ki-67 labeling index or mitotic counts) into well-differentiated (WD) NETs (G1–G3) and poorly differentiated (PD) NECs (always G3) (Table 1). Based on clinical and pathological evidence, an additional tumor category characterized by high proliferative capacities despite WD morphology (NET G3) was incorporated first in the 2017 WHO classification of pancreatic NENs and then in the 2019 WHO classification of digestive NENs [10]. Additional immunohistochemical markers, such as Rb lost and p53 mutant pattern in pancreatic NECs compared with loss of ATRX, DAXX, and menin in panNETs, can help in separating these two entities [8, 9] (III, B).

**Table 1:** WHO classifications of gastroenteropancreatic and lung neuroendocrine neoplasms

2019 WHO classification of gastroenteropancreatic neuroendocrine neoplasms					2015 WHO classification of pulmonary neuroendocrine neoplasms	
Terminology	Differentiation	Grade	Mitotic count (2 mm^2^)^a^	Ki-67 index (%)^a^	Terminology	Criteria
Neuroendocrine tumor G1	Well differentiated	G1	< 2	< 3	Typical carcinoid	Carcinoid morphology < 2 mitoses/2 mm^2^ No necrosis
Neuroendocrine tumor G2	Well differentiated	G2	2–20	3–20	Atypical carcinoid	Carcinoid morphology 2–10 mitoses/2 mm^2^ Necrosis (often punctuate)
Neuroendocrine tumor G3	Well differentiated	G3	> 20	> 20	Large cell neuroendocrine carcinoma	≥ 11 mitoses/2 mm^2^ (median 70/2 mm^2^) Necrosis (often large zones) Cytologic features of NSCLC
Neuroendocrine carcinoma Small cell type Large cell type	Poorly differentiated	G3	> 20	> 20	Small cell neuroendocrine carcinoma	≥ 11 mitoses/2 mm^2^ (median 80/2 mm^2^) Necrosis (often large zones) Cytologic features of SCLC
Mixed neuroendocrine/non-neuroendocrine neoplasm (MiNEN)^b^	Well or poorly differentiated	Variable				

*WHO* World Health Organization, *NSCLC* non-small-cell lung cancer, *SCLC* small-cell lung cancer

^**a**^The fnal grade is determined based on whichever index (mitotic count or Ki-67 index) places the tumor in the highest grade category

^b^Digestive MiNEN are neoplasms in which the two components are morphologically and immunohistochemically recognizable and each of them represents at least 30% of the tumor

The 2015 WHO Classification of lung NENs [11] recognizes four histologic variants: TC, AC, large-cell neuroendocrine carcinoma (LCNC) and small-cell neuroendocrine carcinoma (SCNC), according to the morphological differentiation, mitotic count, and presence/absence of necrosis (Table 1). In contrast to the GEP-NENs’ classification, Ki-67 index is not required for tumor grading. Similarly to G3 GEP-NET, lung NENs with WD morphology but proliferative capacities higher than those accepted for TC and AC (mitotic count > 10/2 mm^2^ and/or Ki-67 index > 20%) are becoming increasingly recognized in the literature. This category does not formally exist in the current thoracic WHO classification, which provisionally suggests classifying such tumors as “LCNEC with morphologic features of carcinoid tumor” [12].

The recently proposed 2022 WHO Classification of Endocrine and Neuroendocrine Tumors includes for the first time NENs of non-endocrine organs, as well as some modifications and relevant features applied to NENs. When adopted into clinical practice, it will be an important step forward to ensure accurate diagnosis and form the basis of future research in this field [9].

### Staging and risk assessment

Besides the histopathological diagnosis, the following procedures are recommended for an adequate characterization and staging of NENs [13]:

- Biochemical markers: Chromogranin A (III, B), 24-h urine 5-hydroxyindoleacetic acid (5-HIAA) in metastatic SI-NETs regardless of symptoms and lung tumors with carcinoid syndrome (III, A), specific hormones in pancreatic or lung NENs presenting with suspected hormonal syndromes (e.g., insulin for insulinomas, glucagon for glucagonomas, cortisol/ACTH for Cushing syndrome) (III, A) [13].
- Anatomic imaging: Computed tomography (CT) constitutes the basic radiological study for the assessment of location and extent of disease. NETs are generally hypervascular and best visualized in the late arterial phase. Triphasic helical CT of the abdomen and pelvis should therefore be used for the optimal evaluation of liver metastases. Chest CT is also recommended to study lung metastases (III, A). Magnetic resonance imaging (MRI), particularly contrast-enhanced MRI utilizing hepatocellular phase-contrast agents, should be preferred compared with CT for the detection of small liver metastases, pancreas, and bone lesions as a result of its higher sensitivity (III, A) [14].
- Functional imaging: Because most NETs overexpress high-affinity somatostatin receptors (SSTR), SSTR-based imaging should be part of the initial staging (II, A). Gallium-68 (^68^Ga)-DOTATATE PET/CT has become the preferred modality for SSTR imaging as a result of its higher sensitivity for baseline whole-body staging, detecting of small lymph-node or bone metastases, and identification of the primary site in cases of occult origin (II, A) [14, 15]. SSTR scintigraphy can be used if ^68^Ga PET/CT is not available, although is considerably less sensitive (III, B). ^18^F-fluorodeoxyglucose PET/CT (FDG-PET/CT) may be considered to refine prognosis in high G2 and G3 NENs, which generally have less SSTR expression and higher glucose metabolisms than low-grade NETs, negative SSTR imaging or rapidly growing disease (III, B) [14].
- Additional diagnostic recommendations vary by disease site and stage and include endoscopic procedures, brain CT/MRI in high-risk lung or NT-pro-BNP and echocardiogram for the evaluation of carcinoid heart disease in SI, lung, and thymic with carcinoid syndrome tumors (III, A) [13].
- Genetic risk evaluation and testing for hereditary endocrine neoplasia syndromes, such as multiple endocrine neoplasia type 1, neurofibromatosis type 1, tuberous sclerosing complex, or Von Hippel Lindau syndrome, must be performed when there is a well-founded suspicion (III, A) [13].

Disease stage and tumor grade are the two major independent prognostic parameters in NENs and should always be assessed (III, A). For staging, the current 8th edition (2017) of the American Joint Committee on Cancer/Union for International Cancer Control (AJCC/UICC) TNM staging system, although not widely adopted, has been proposed for WD GEP-NETs and includes separate classifications for stomach, duodenum/ampulla of Vater, jejunum/ileum, appendix, pancreas, and colorectal primary sites. The 8th edition TNM classification is also recommended for lung NENs even if not specific. For all NECs, the staging classifications of adenocarcinomas must be applied [16].

## G1–G3 neuroendocrine tumors (NETs)

### Management of local and locoregional disease

Surgical resection of the primary tumor should always be considered and discussed at the multidisciplinary team [17]. However, depending on the primary tumor location, tumor prognostic features, and patient-related factors more conservative or aggressive therapeutic decisions could be taken.

An arduous debate can be found in the literature about the pros and cons of what to do in those cases with gastric and pancreatic G1 and G2 NETs between 10 and 20 mm in diameter. Endoscopic submucosal dissection (ESD) and enucleation are considered standard techniques for gastric and pancreatic tumors, respectively, if feasible. Gastroduodenal G1 NETs less than 1.5 cm can be safely treated with endoscopic resection based on general good prognosis. It is widely accepted that pancreatic G1, non-functioning and non-ampullary NETs with less than 10 mm size could be safe to follow a “watch and wait” strategy with a later resection in case there is a significant increase in size up to 20 mm or onset of symptoms. For non-resected small pancreatic incidentalomas, 6 month MRI follow-up can be offered. For G1 or G2 gastric and pancreatic lesions greater than 20 mm, a surgical resection should be discussed with the patient.

Surgery of localized and locally advanced SI-NETs, including systematic lymphadenectomy and accurate evaluation of the entire intestine, is recommended as long as it is associated with improved outcomes.

Those NETs with a periampullary location should be faced by radical surgery plus lymphadenectomy unless the primary tumor size is less than 15 mm, where ampullary resection alone could be considered. NETs from the appendix are generally incidentally found in an appendectomy and in case that primary tumor exceeds 20 mm or shows poor prognostic features (mesoappendix invasion, lymphovascular infiltration, and G2) or atypical histology like MiNEN (mixed-neuroendocrine neoplasm), an additional partial colectomy with lymphadenectomy should be performed [18].

Colonic primary NETs should be approached as adenocarcinomas in terms of surgical procedures. Total colonoscopy is mandatory for patients with rectal NETs to discard other colorectal NETs that can occur in up to 8% of cases. For rectal NETs with < 15 mm, endoscopic mucosal resection (EMR) or ESD should be offered. In cases with an initial R1 endoscopic resection, transanal endoscopic microsurgery should be considered.

Resection of WD primary functional NET may improve survival in patients with liver metastases and should be discussed if feasible in the multidisciplinary team [19]. Primary tumor resection in non-functional asymptomatic stage IV SI-NETs is still a matter of debate [20].

Surgery is rarely curative for those patients with NECs and in the few cases where the disease is diagnosed in a localized stage. Although evidence of benefit is lacking in the absence of prospective clinical trials, adjuvant treatment based on platinum and etoposide may be considered after surgery given the high rate of recurrence [21].

Diffuse idiopathic pulmonary neuroendocrine cell hyperplasia (DIPNECH) is a rare pulmonary condition, characterized by diffuse proliferation of neuroendocrine cells in the respiratory epithelium. DIPNECH lesions are less than 5 mm in size and are limited to the basement membrane with no invasion. Currently, the management and treatment options for DIPNECH include clinical observation, oral and inhaled steroids, somatostatin analogs, chemotherapy, surgical lung resection, and even lung transplantation [22].cT1N0 bronchial NETs may benefit from an initial radiological follow-up without treatment (mainly if measure less than 10 mm) to determine the growth rate. In case of onset of clinical syndrome or radiological progression, sublobar surgical approaches including wedge resection should be considered [6].

Adjuvant treatment is generally accepted for aggressive large and SCNCs; however, it is less well established for pulmonary carcinoids, for which NETs guidelines admit a lack of trials to support a high-level recommendation for adjuvant therapy.

The use of locoregional techniques like transarterial embolization (TAE), transarterial chemoembolization (TACE), cryotherapy, and selective internal radiation therapy (SIRT) to control the growth of both localized tumor and distant metastasis that cannot be achieved with surgery can be considered as an alternative approach to systemic therapies, especially for patients with symptomatic/functioning high liver tumor burden. Using these local techniques, up to 80% of clinical responses have been reported. [13].

**Recommendations:**

- Locoregional approach to a primary NET should be systematically discussed in the multidisciplinary team [III, A].
- Pancreatic and gastric G1 non-functioning NETs with less than 10 mm can be followed using a “watch and wait” strategy [III, A].
- Surgical resection should be discussed with the patients for gastric and pancreatic tumors greater than 20 mm [III, A].
- NETs with a periampullary location should be faced by radical surgery plus lymphadenectomy if size is greater than 15 mm [III, B].
- aNETs that exceed 20 mm, present with mesoappendix infiltration, lymphovascular invasion, or high tumor grade should be considered for additional colectomy with lymphadenectomy [III, B].
- Lung carcinoids over 10 mm, functioning ones or those that experience progression, should be considered for sublobar surgical approaches including wedge resection [III, A].

### Management of metastatic disease

#### Surgery and liver-directed therapies (Fig. 1)

**Fig. 1:**
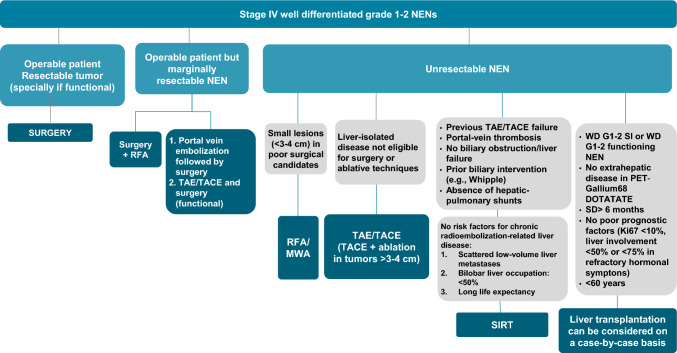
Algorithm of surgery and liver-directed therapies. *NEN* neuroendocrine neoplasm, *RFA* radiofrequency ablation, *MWA* microwave ablation, *RF* radiofrequencies, *TAE* transarterial embolisation (con z), *TACE* transarterial chemoembolization, *SIRT* selective internal radiation therapy, *WD* well differentiated, *G* grade, *SD* stable disease

Resection of primary small bowel tumors in the setting of unresectable metastases has no confirmed survival benefit but could be contemplated to reduce the risk of bowel obstruction or ischemia. In the absence of randomized trials, different cohort studies suggest that surgery for liver metastases in WD NEN may be superior to nonsurgical therapeutic approaches, achieving a 5-year OS of 75%–100% versus 35–65% [23]. Classically, a liver debulking threshold above 90% has been considered the optimal criterion. However, some authors suggest that cytoreduction above 70% is sufficient to improve survival and relieve symptoms in WD NEN [24]. The use of intravenous octreotide during surgery (50 μg/h for 24 h) could be useful to minimize the risk of carcinoid crisis [25].

Given the multifocal and often bilobar distribution of liver metastases, only 20% of patients with WD NEN are suitable for complete resection, with functional reserve volumes estimated of at least 20% [23]. Patients with WD NEN and unresectable liver metastases may be candidates for intra-arterial liver therapies due to hypervascularity of the metastases, assuming well-preserved liver function and good general condition, although no randomized trials are available. These techniques may complement or be an alternative approach to systemic therapies, especially for patients with a high symptomatic/functional liver tumor burden, due to the high rate of symptom control. For transarterial embolisation (TAE) and transarterial chemoembolization (TACE) with doxorubicin or mitomycin C, radiographic response rates were 50.7% and 38.9%, symptomatic response rates, 60% and 47% and PFS around 20 months, respectively, in a systematic review [26]. Addition of intra-arterial chemotherapy (TACE) could improve outcomes in pancreatic NENs [27]. TAE can achieve symptom control allowing for subsequent definitive resection in patients with severe hormonal syndrome with nutritional compromise [28]. Selective internal radiation therapy (SIRT) with yttrium-90 (90Y)-labeled glass or resin microspheres is increasingly used due to an apparent favorable side-effect profile and the need for fewer treatment sessions compared to TAE/TACE. In a retrospective international multicenter study (*n* = 244), the safety and efficacy of SIRT was demonstrated even in patients with high liver tumor burden and disease progression after the previous therapies [29]. According to a meta-analysis (27 series), SIRT provides 51% ORR, 88% DCR, and a median OS of 32 months [23]. Contraindications for SIRT include the presence of hepatopulmonary shunts (not to exceed 610 MBq, equivalent to approximately 30 Gy/single treatment, in the mapping with technetium-99 macro aggregated albumin), uncorrectable reflux from the hepatic artery to the stomach, pancreas or bowel, and severe pulmonary dysfunction.

Laparoscopic or percutaneous ablations [microwave, laser, or radiofrequency ablation (RFA), and cryotherapy] are useful techniques for the treatment of small liver metastases, in combination with resection or in case of recurrence after liver surgery. Its advantages are low morbidity and mortality and symptomatic control, while the main disadvantage is the almost universal local recurrence rate. Limitations include poor ablation efficacy in tumors > 3–4 cm. The proximity (< 1 cm) to central biliary structures or portal veins usually constitutes a contraindication as the resulting heat can lead to biloma, portal thrombosis, and death [28].

Although the evidence is scarce, liver transplantation has been considered an alternative for WD G 1 and 2, SI or functioning NEN, with stabilization > 6 months with the previous treatment, without poor prognostic factors, after assessing the unfeasibility of other approaches, given that 5-year OS is 69–97.2% in highly selected patients [30].

In short, surgery is the therapeutic alternative of choice for resectable liver metastases. SIRT or TAE/TACE could be indicated in unresectable liver-predominant metastases especially in functioning tumors with RFA being an alternative in small metastases (III, B) and liver transplantation only in highly selected cases (III, C).

#### Specific treatment of hormone-related syndromes (Fig. 2)

**Fig. 2:**
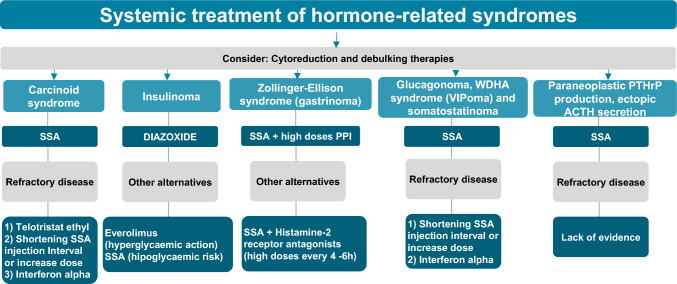
Systemic treatment of hormone-related syndromes. *SSA* somatostatin analogs, *PPI* proton-pump inhibitors, *WDHA* watery diarrhea, hypokalaemia, achlorhydria, *PTHrP* parathyroid hormone-related protein, *ACTH* adrenocorticotropic hormone

Functional NENs are associated with a wide range of hormone excess syndromes with dermatological, endocrine, gastrointestinal, and cardiovascular symptoms. The presence of hepatic metastases favors the release of active hormones into the hepatic venous circulation and their entry into the systemic circulation before hepatic metabolism, leading to hormone hypersecretion syndromes [31]. The use of SSA is the cornerstone of symptomatic management of GEP-NENs with rapid clinical relief from the first injections (with subsequent improvement and stabilization since the fourth dose) [31]. SSAs decrease diarrhea (up to 75%) and flushing (up to 81%) and control symptoms in more than 90% of patients with glucagonomas, VIPomas, and somatostatinomas, 80% of those with gastrinomas, typical and atypical carcinoid syndrome, and 50% of insulinomas [31]. However, SSAs can aggravate hypoglycemia and inhibit counter-regulatory hormones in patients with insulinoma, as half of them do not express SSTR2, with diazoxide (3–8 mg/kg/day in several doses with meals) being the drug of choice in these cases. In gastrinomas, the cornerstone of symptomatic treatment is high-dose proton-pump inhibitors with more unpredictable antisecretory activity of SSA [32]. Cytoreduction strategies, including surgery or intrahepatic local therapies, are an adjunctive treatment in functioning NENs, and should be considered whenever possible, especially in highly symptomatic cases [23]. Other rare hormonal syndromes, such as paraneoplastic parathyroid hormone-related protein (PTHrP) production, ectopic ACTH secretion, and osteomalacia due to Cushing's syndrome, may also improve with SSA [31].

In very symptomatic patients, short-acting octreotide 500 µg/day in 2 doses during the first 2 weeks, increasing individually until symptom control, may contribute to faster symptom relief [31]. Up to 20–40% of functioning NENs become refractory to SSA, and other targeted therapies such as everolimus (e.g., in insulinoma), chemotherapy, or PRRT may contribute to clinical improvement. As a specific treatment, telotristat ethyl has shown activity in the control of refractory carcinoid diarrhea in patients with ≥ 4 bowel movements per day with SSA (RCT phase III TELESTAR) and in patients with less severe carcinoid syndrome (RCT phase III TELECAST) with positive impact on quality of life [33, 34]. Therefore, telotristat ethyl (250 mg t.i.d.) is indicated for diarrhea associated with carcinoid syndrome in patients insufficiently controlled with SSA. Interferon α2a has been tested alone and in combination with SSA to optimize tumor response and overcome SSA resistance without conclusive results and with low-quality studies. Nevertheless, different trials have reported clinical response rates of 50–70% with interferon alone or in combination with SSA. Therefore, interferon may be an alternative second-choice treatment option when SSAs fail due to their unfavorable adverse effect profile [31]. Another alternative when symptoms become refractory is to modify the SSA schedule: (1) dose escalation, although there is some evidence that SSTRs can saturate with an octreotide LAR dose of 60 mg or more, so higher doses may be associated with marginal benefits; (2) shortened interval from 28 to 21 or 14 days (phase II CLARINET-FORTE trial) [35]; (3) octreotide or lanreotide as an extra rescue dose, or 4) switch to a continuous octreotide pump.

Ultimately, SSA is the treatment of choice in unresectable metastatic functioning NEN (I, A).

## Antiproliferative

**Fig. 3:**
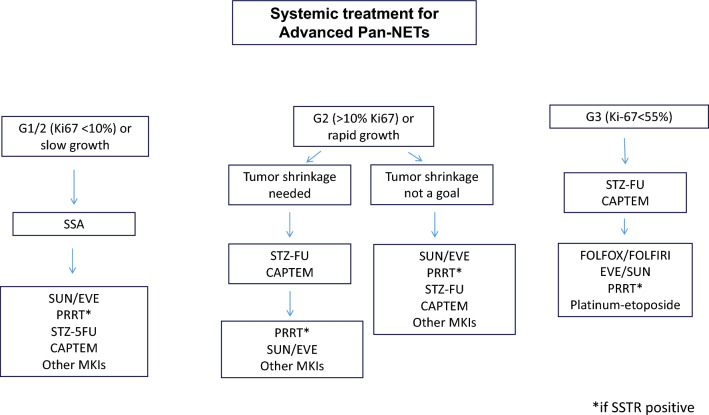
Systemic treatment for advanced pan-NETs

**Fig. 4:**
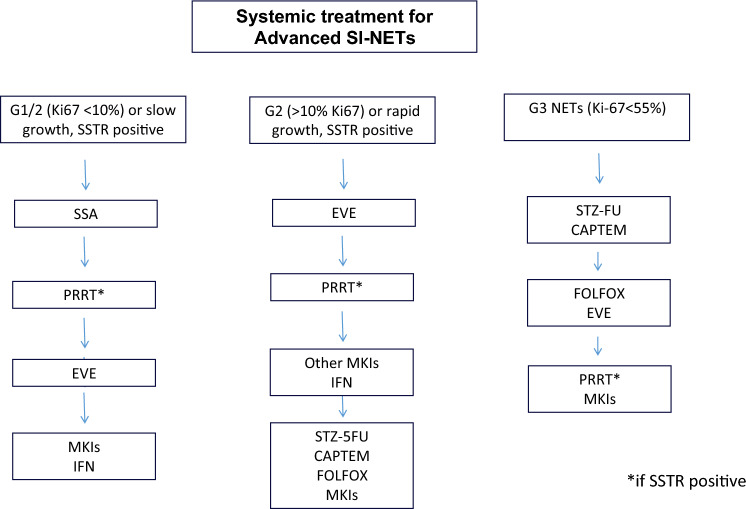
Systemic treatment for advanced SI-NETs

**Fig. 5:**
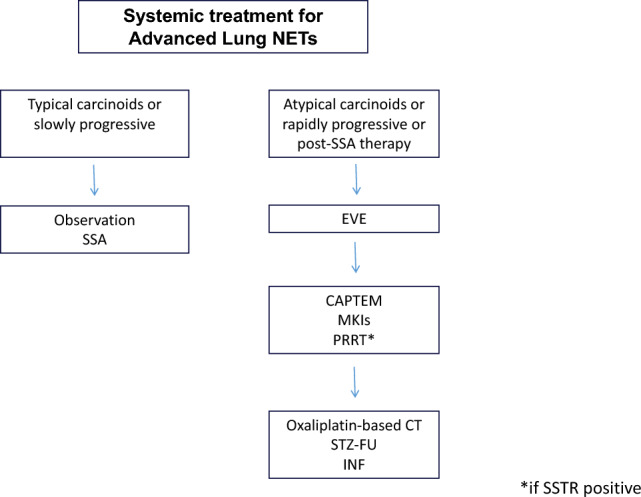
Systemic treatment for advanced lung NETs

Options of treatment with an antiproliferative effect for patients diagnosed with WD NETs have grown over the last decade, with practice now based on randomized phase III evidence for most of treatment options (Figs. 3, 4, 5).

### Somatostatin analogs (SSA)

There are two clinical trials supporting the antiproliferative effect of SSA in midgut (octreotide and lanreotide, IA) and pancreatic (lanreotide, IA) NETs. The PROMID study enrolled 85 patients with G1 metastatic midgut NETs, which were randomized to receive octreotide LAR (30 mg/28 days) or placebo. Time to tumor progression was significantly longer in the octreotide LAR group (14.3 months) as compared to patients treated with placebo (6 months) [HR 0.34, 95% confidence interval (CI) 0.20–0.59, *p* < 0.001]. The greatest effect was observed in patients with low hepatic tumor load and resected primary tumor [36]. No difference was observed, however, in OS among study arms. Thereafter, the CLARINET study included 204 patients with non-functioning GEP-NETs and ki67 < 10% (45% pancreatic, 36% midgut, 7% hindgut, and 13% of unknown primary), that were randomly allocated to receive lanreotide autogel (120 mg/28 days) or placebo [37]. Treatment with lanreotide significantly prolonged progression-free survival (PFS) over placebo (median not reached with lanreotide vs. 18 months with placebo, HR 0.47, *p* < 0.001). Of note, patients with high liver involvement (> 25%) also benefited from lanreotide. Currently recommended antiproliferative doses are octreotide LAR 30 mg IM or lanreotide autogel 120 mg sc every 28 days. Lower starting doses may be used for syndrome control, and then be titrated as needed. Adverse effects of SSA include malabsorption, hypo or hyperglycemia, hypothyroidism, pain, and erythema at the site of injection, hypersensitivity reactions, and cholelithiasis on long-term use. Careful monitoring of the development of pancreatic exocrine insufficiency is recommended [38].

The antiproliferative impact of lanreotide for bronchial NETs is supported by the phase III SPINET randomized clinical trial [39]. Recruitment stopped early due to slow accrual [SSA used as standard of care (SOC) in many countries], when a total of 51 and 26 patients had been randomized to SSA and placebo, respectively. The use of lanreotide 120 mg every 4 weeks was associated with a median PFS of 16.6 months. PFS was greater in TC (median PFS 21.9 months) that in AC (median PFS 14.1 months), as expected. Based on these results, lanreotide could be utilized with an antiproliferative aim in bronchial NETs (II, B).

The antiproliferative effect of high doses of SSA in GEP-NETs (lanreotide 120 mg every 2 weeks) after radiological progression to standard-dose SSA was tested in the phase II CLARINET-FORTE study [35]. A total of 99 patients were recruited (51 in the midgut cohort; 51 in the pancreatic cohort). Median PFS were 8.3 (95% CI 5.6–11.1) and 5.6 (95% CI 5.5–8.3) months, respectively. Median PFS were notably higher in patients with Ki-67 ≤ 10% in both cohorts (8.6 and 8.0 months in midgut and panNETs, respectively), whereas rapid progression was observed in those with Ki-67 > 10% regardless of primary site (median PFS of 5.5 and 2.8 months, respectively). Based on these data, increasing lanreotide dose could be considered in selected patients if other treatment options (less well tolerated) may not be an option due to patient´s characteristics, particularly in those with lower proliferative index or slow growing tumors (III, B).

With the lack of prospective data demonstrating benefit of SSA in combination with other therapies after progression to SSA monotherapy, they should only be maintained in functioning tumors to facilitate hormonal-related symptoms (III, C).

### Interferon

Interferon may have some antiproliferative activity and also symptomatic control activity for functioning tumors (mainly carcinoid syndrome), although this has not been definitively proven [40], and it is therefore generally indicated after failure of other therapeutic options (II, C).

### Targeted agents

Sunitinib has demonstrated in an international, placebo-controlled phase III clinical study, significantly increased PFS in patients with advanced panNETs (median PFS: 11.4 vs. 5.5 months; HR 0.42; 95% CI 0.26–0.66, *p* < 0.001) [41] (I, A). The results obtained in this trial led to the approval of sunitinib by regulatory authorities for the treatment of advanced and progressive, well-moderately differentiated, functioning, and non-functioning panNETs. The RADIANT-3 trial randomized patients with advanced well-moderately differentiated panNETs to receive everolimus or placebo demonstrating an increase of PFS in favor of everolimus (11.0 vs. 4.6 months; HR 0.35; 95% CI 0.27–0.45, *p* < 0.001) [42]. The RADIANT-4 trial confirmed everolimus effectiveness in non-functioning NETs of lung or gastrointestinal origin. PFS was significantly increased with everolimus as compared to placebo (11.0 vs. 3.9 months, respectively, HR 0.48; 95% CI 0.35–0.67, *p* < 0.00001) [43]. In contrast, the effect of everolimus on PFS failed to achieve statistical significance in the RADIANT-2 study conducted in functioning midgut NETs [44]. The results obtained by the RADIANT-3 and -4 trials led to the approval of everolimus by regulatory authorities for the treatment of advanced and progressive, well-moderately differentiated, functioning and non-functioning panNETs, and for non-functioning gastrointestinal and lung NETs (I, A).

Several other MKIs, mainly targeting angiogenesis, have also been assessed in NETs. For midgut NETs, lenvatinib reported a radiological response rate (RR) of 16.4%, with disease control rate of 92.7% and a median PFS of 15.7 months (95% CI 12.1–19.5) in the phase II TALENT-GETNE study (IIIB) [45]. Surufatinib was tested in a randomized phase III study including non-panNETs in China [46] and showed a median PFS of 9.2 months in the surufatinib arm vs 3.8 months with placebo, HR 0.33 (95% CI 0.22–0.50); *p* < 0.0011 (IIB). Data in non-Chinese patients are limited, but similar pharmacokinetic and toxicity profile is suggested in a phase I/II study conducted in the USA in heavily pretreated patients [47]. Currently, a phase II study is being conducted in Europe. Axitinib combined with octreotide was evaluated in 256 non-panNETs in the context of the randomized double-blind placebo-controlled phase II/III AXINET-GETNE study [48]. The study did not meet statistical significance for the investigator-assessed PFS (primary endpoint) (median 17.2 (axitinib) vs 12.3 (placebo) months, HR 0.816, *p* = 0.169). However, PFS per blinded independent central review was superior in the axitinib arm (median PFS of 16.6 vs 9.9 months; HR 0.687, *p* = 0.01) (IIB) [49]. Cabozantinib is currently being tested in a phase III study [CABINET (NCT03375320)], following promising activity in a phase II study (41 midgut NETs, RR of 15%, median PFS of 31.4 months [95% CI 8.5-not reached)] [50].

For panNETs, lenvatinib reported a radiological RR of 44.2%, with median duration of response of 19.9 months and a median PFS of 15.6 months in the phase II TALENT study (IIIB) [11]. Surufatinib also showed PFS benefit in panNETs in a randomized phase III Chinese study (ORR of 19% and median PFS of 10.9 months in the surufatinib arm vs 3.7 months with placebo, HR 0.34 (95% CI 0.21–0.55); *p* < 0.0001) (IIB) [51]. Cabozantinib is also being explored in PanNETs in a phase III study (CABINET trial, NCT03375320) following promising activity in a phase II study (partial RR of 15% in 20 patients with PanNETs with favorable toxicity profile) [50].

### Immunotherapy

Immunotherapy approaches in NETs have been disappointing. Monotherapy has not really shown much activity [52]. Combination strategies of dual immunotherapy compounds, such as durvalumab and tremelimumab (DUNE-GETNE study), have been explored but reported limited activity in NETs [53]. Thus, immune checkpoint inhibitors are unlikely to play a major role in the treatment of WD NETs.

### Chemotherapy

Systemic chemotherapy is indicated in panNETs (mainly in the presence of high G2, progressive or bulky advanced disease) and G3 NENs (both NETs and NECs). Current evidence for the use of chemotherapy is stronger for pancreas (II, B) than for midgut (only to be used in very selected scenarios with rapidly progressive disease and failure of other treatment strategies; III, C) or lung NETs (III, C). If chemotherapy is to be considered, both streptozocin with 5-fluorouracil (STZ-5FU) (II, B) or temozolomide and capecitabine (II, B) are the preferred treatment options. Selecting one or another will rely on convenience, availability, patient wishes, and compliance. Other options include 5FU-based chemotherapy regimens (i.e., FOLFOX).

For panNETs, classical chemotherapy combinations include streptozotocin (STZ) with either 5-FU or doxorubicin, with overall RR of 45–69% in older trials (II, B) and 28–42% in more recent ones, and PFS ranging from 16 to 23 months [54]. Very recently, preliminary results from the phase III SEQTOR-GETNE1206 trial have been reported [55]. This trial randomized 140 patients with progressive panNETs to receive two different treatment sequences, everolimus followed by STZ-5FU upon disease progression or the reverse sequence. RR with first-line therapy was significantly greater in patients treated upfront with chemotherapy than in those that received everolimus (30% vs 11%, *p* < 0.05), with no significant differences in PFS1 among study arms (21.5 vs 23.6 months, *p* = 0.33). These results suggest chemotherapy should be preferred when tumor shrinkage is an important treatment goal. Also, recently updated results of a randomized phase II trial (E2211) that compared temozolomide and capecitabine to temozolomide alone in panNETs (*N* = 133) showed an increase in PFS (from 14.4 to 22.7 months, HR 0.58, *p* = 0.022) with no significant impact in OS (53.8 vs 58.7 months, HR 0.82, *p* = 0.42) and similar RRs (34% vs 40%) for the combination as compared to monotherapy (II, B) [56, 57]. ORR were significantly higher in a subset of patients included in this trial with MGMT deficiency assessed by immunohistochemistry (*N* = 97) (52% vs 15% in MGMT low vs high) or promoter methylation (*N* = 57) (85% vs 38% in patients with or without methylation), with a non-significant positive trend toward improved PFS and OS [57]. This trial, however, was not designed to assess the predictive role of MGMT deficiency as both arms contained temozolomide. Therefore, assessment of MGMT is not considered standard in routine clinical practice.

No site-specific randomized studies have been performed in midgut and lung NETs (typical and atypical carcinoids). Temozolomide-based regimens have been explored in non-randomized studies as well as oxaliplatin combinations, with PFS ranging from 5 to 20 months [58] (III, C).

For management of G3 NETs, adenocarcinoma-like, alkylating-based chemotherapies may be the most effective treatments, in terms of RR and PFS, with etoposide-platinum chemotherapy showing poor efficacy (IIIC) [59].

### Peptide receptor radioligand therapy (PRRT)

Patients with advanced disease and a positive SSTR imaging may be considered for peptide receptor radionuclide therapy (PRRT). PRRT primarily utilizes one of two radioisotopes, 90 Yttrium (90Y) or 177 Lutetium (177Lu), linked to an SSA via the chelating agent 1,4,7,10-tetraazacyclo-dodecane-1,4,7,10-tetraacetic acid (DOTA).

The phase III NETTER-1 study, conducted in patients with midgut NETs progressive to standard-dose SSA, has shown that Lutetium (177Lu)-DOTATATE, compared to high doses of octreotide, significantly increases the RR (18 vs. 3%; *p* < 0.001) and PFS (28.4 vs 8.4 months; HR 0.21, 95% CI 0.14 to 0.33, *p* < 0.0001) [60], with no significant improvement of OS (48.0 vs 36.3 months, HR 0.84, 95% CI 0.60–1.17, *p* = 0.30) [61]. In addition, 177Lu-DOTATATE was able to improve the subjective measurement of patient's quality of life in multiple relevant domains, such as global health status, physical functioning, role functioning, fatigue, diarrhea, pain, disease-related worries, and body image [60]. The most common grade 3–4 toxicities were lymphopenia (9%), and emesis (7%) with no significant renal toxicity (mean change from baseline in creatinine clearance over time was similar for both study arms) [60]. With a median follow-up of over 76 months, 2 of 111 patients (1.8%) treated with 177Lu-DOTATATE developed a myelodysplastic syndrome; one of whom died 33 months after randomization.

The role of PRRT in non-midgut NETs relied on retrospective series until 2022 [62, 63] when the first prospective study exploring PRRT in panNETs was reported. The phase II OCLURANDOM study randomized progressing panNET patients to sunitinib or PRRT [64]. Within the 84 patients enrolled, it is worth noticing that a significant proportion of patients had received additional prior systemic therapy other than SSA (including chemotherapy in more than 50% of cases in each arm). The study met its primary endpoint with improved 12-month PFS rate (80.5% vs 42.0% in favor of the PRRT arm). Median PFS was 20.7 months in the PRRT arm (90% CI 17.2–23.7) and 11 months in the sunitinib arm (90% CI 8.8–12.4).

Therefore, PRRT is approved in patients with WD, metastatic, unresectable gastroenteropancreatic NETs with positive SSTR (grade 2–4 Krenning scale) with stronger evidence in midgut (I, A) than in pancreatic origin (II, B). Its administration to tumors of other primary sites may also be considered although the evidence to support it is not derived from randomized trials (III, B). The appropriate timing of this therapeutic intervention remains to be elucidated.

## Extrapulmonary neuroendocrine carcinomas

### Introduction

In the WHO 2019 classification, there are two subtypes of NECs, small and large cell, both with mitotic count > 20/2 mm^3^ and ki67 index > 20%. G 3 NECs frequently develop metastatic disease after the initial diagnosis at localized stage [10]. Gastrointestinal tract is the most common site for extrapulmonary NECs (35–55% of all). NECs’ classification based on proliferation ki67 index has shown to have implications in response to chemotherapy and prognosis: patients with ki67 ≥ 55% tumors respond better to platinum but have a worse prognosis. Evidence to treatment recommendations for NECs derives from limited controlled clinical trials and small non-controlled studies. Clinicians may tend to treat this entity in analogy to small-cell lung cancer due their histological and clinical resemblance. Nevertheless, principles of chemotherapy for small-cell lung cancer should not be extrapolated to gastrointestinal extrapulmonary NECs.

### Localized disease

Localized disease is usually managed with surgery. However, retrospective series indicate that this approach alone is rarely curative and suggest improved results with adjuvant chemotherapy despite no single prospective clinical trial evaluated this strategy in extrapulmonary NECs [65, 66] (IV, C). Radiotherapy may be used as consolidation therapy in this scenario, and as an alternative to surgery in certain primary sites (i.e., esophageal NECs) [67] or in the setting of non-resectable locally advanced disease (IV, C).

### Metastatic disease

Systemic chemotherapy with palliative intent is the mainstay of care of patients with metastatic NEC. Principles of chemotherapy for lung SCNC should not be extrapolated to GEP-NECs. Initial treatment should include a two-drug platinum regimen: cis or carboplatin plus etoposide, with no well-established number of cycles, so that treatment until progression or unacceptable toxicity may be considered [68, 69]. Irinotecan plus cisplatin is also an acceptable regimen [70]. The addition of immune checkpoint inhibitors targeting PD-1 or PD-L1 to first-line chemotherapy in extrapulmonary NECs is under evaluation [71]. No standard treatment has been established in second-line setting: temozolomide-based regimens (Ki67 index 20–55% and platinum refractory disease) [72], FOLFIRI [73] or FOLFOX [74], and topotecan [75]-based chemotherapy have been explored. More recently, liposomal irinotecan-FU combination, docetaxel [76], as well as immunotherapy have also been studied [53, 77]. Patients with sensitive platinum disease (progression 3–6 months after the end of first-line treatment) may benefit from re-treatment. Doublet regimen of cetuximab/encorafenib could be considered in refractory platinum patients colorectal NECs harboring BRAF V600E mutation, as formal approval for this combination is extensive to all colorectal carcinomas. The addition of bevacizumab to chemotherapy does not add any benefit and should not be recommended [78].

Immune checkpoint inhibitors alone and in combination have been evaluated in large phase II studies and showed limited activity in extrapulmonary NECs. The initial reports with high response rates (44%) with nivolumab and ipilimumab [79] have not been confirmed in the French and Spanish prospective studies NIPINEC [77] and DUNE [53] studies (objective responses 9–11%). However, some prolonged survivals were observed without any predictive biomarker for patient selection and deserves further evaluations.

Surgical metastasectomy is not recommended in NECs, although multidisciplinary decision should be individualized in selected patients with liver only disease.

In conclusion for metastatic disease, initial treatment should include two-drug platinum regimen plus etoposide or irinotecan (II, B). No standard treatment has been established in second line (IV, C).

## Follow-up, long-term implications, and survivorship

The main rationale for the long-term follow-up in NETs is the increase in cumulative recurrence rate even 10 years after surgical resection [77]. The relapse rate in lung NET ranges between 4 and 26%, in SI-NET between 23 and 58% and in panNET between 12 and 69% [80, 81]. Nevertheless, there is no clear guidance on follow-up as no high-quality evidence-based recommendations are available.

Clinical follow-up is recommended every 3–12 months within the first 3 years in patients with resected G 1/2 NET and every 3 months in patients with G 3 [82–84] (V, C). Time intervals may extend to 1–2 years with increasing length of follow-up. Cross sectional imaging should be included in the follow-up and CT, the preferable option, can alternate with MRI or, in particular cases, ultrasonography (US) (V, C). SSTR imaging is not fully recommended during follow-up, but when performed, it should be done according to presurgical positive findings or every 2 years in G 1 or 2 tumors [82, 83] (V, C). For patients with advanced disease, indefinitely monitoring is required every 3–6 months according to tumor characteristics and response to treatment [82, 83] (V, C).

Despite their limited value, serum markers are generally recommended during follow-up in patients with elevated baseline values. Those markers are chromogranin A in NET or neuron-specific enolase (NSE) in PD NEC (V, C). Other serum peptides or urine 5-HIAA are recommended in patients with suspicious symptoms of disease recurrence from a functioning tumor or in the metastatic setting [80–83] (V, C). Endoscopy is also recommended when recurrence is clinically suspected [83] (V, C). In patients with carcinoid syndrome and elevated 5-HIAA, echocardiography and NT-pro-BNP are recommended at baseline and, thereafter, yearly, or according to clinical symptoms (V, C).

Some patients may not need further follow-up, such as those with completely resected < 2 cm, G 1/2 appendiceal NET [83] (V, C).

Long-term survival has been reached in patients with resected and, even, unresectable NEN. This achievement highlights the need of focusing on nutritional status, physical exercise, management of adverse events or sequelae from the different treatment strategies, psycho-social resources, and well-being for a better quality of life [85].
